# Impaired K48-polyubiquitination downmodulates mouse norovirus propagation

**DOI:** 10.3389/fcimb.2025.1530166

**Published:** 2025-05-06

**Authors:** Emmrich Wakeford, Elisabeth Werkmeister, Delphine Cayet, Sabine Poiret, Catherine Daniel, Jason Mackenzie, Jean-Claude Sirard, Frank Lafont, Ghaffar Muharram

**Affiliations:** ^1^ Univ. Lille, CNRS, Inserm, CHU Lille, Institut Pasteur de Lille, U1019 - UMR 9017 - CIIL - Centre d’Infection et d’Immunité de Lille, Lille, France; ^2^ Department of Microbiology and Immunology, University of Melbourne, at the Peter Doherty Institute for Infection and Immunity, Melbourne, VIC, Australia

**Keywords:** norovirus, ubiquitn, innate immunity, signaling, macrophage, inflammation

## Abstract

**Introduction:**

Noroviruses are small non-enveloped, single stranded positive-sense RNA viruses that belong to the family *Caliciviridae*. They are highly contagious and resistant to multiple detergents and are the infectious agents in the majority of viral gastroenteritis in adults. Due to a lack of approved preventive or curative therapy options, intensive research effort is ongoing to better understand the pathogenesis mechanisms of noroviruses.

**Methods:**

In this study, using the persistent murine norovirus S99 strain (MNoV_S99), we have investigated the role in the regulation of anti-noroviral responses of ubiquitination, a post-translational modification that covalently adds one or multiple ubiquitin molecules onto lysine residues of target proteins. To that end, we have first generated RAW264.7 cells overexpressing YFP-Ubiquitin_WT, _K29R, _K48R or_K63R constructs. All non-WT constructs encode a ubiquitin fusion protein with one lysine mutated into an arginine residue, thus preventing the formation of the K29-, K48- or K63-dependent polyubiquitin chains respectively.

**Results:**

Upon infection of these cells with MNoV_S99, we unexpectedly observed that only cells expressing the YFP-Ubiquitin_K48R protein showed a significantly impaired expression of several viral markers: NS5, NS7, VP1 and the replication intermediate dsRNA. Consequently, the number of viral genome copies or viral titers were also significantly decreased in the YFP-Ubiquitin_K48R cells compared to the YFP-Ubiquitin_WT cells. This negative regulation cannot be explained by perturbed viral entry, but rather a constitutive hypersecretion of the pro-inflammatory cytokine TNF and downstream upregulation of IκBα phosphorylation and the subsequent NF-κB nuclear translocation.

**Conclusion:**

Overall, these consequences combined impose a non-permissive environment for MNoV_S99 replication and propagation.

## Introduction

Human noroviruses (HuNoVs) are estimated to be the cause of 80 to 90% of all gastroenteritis of viral origin. In addition to having a high human cost with around 700 million infected people and over 200 000 deaths yearly, norovirus infections are also associated with a very high socio-economic burden (Bartsch et al., 2016). Noroviruses are small non-enveloped, single stranded positive-sense RNA viruses that belong to the family *Caliciviridae* (Robilotti et al., 2015). They are highly contagious due to a low minimal dose of infection (estimated ∼20 virions (Teunis et al., 2008)) and a high resistance to multiple detergents (Zonta et al., 2016). The main hinderance to the successful study of HuNoVs is the lack of efficient cellular models to produce these viruses. Additionally, animal model-based studies with HuNoVs are not available due to a strict receptor dependent species specificity (Haga et al., 2016; Orchard et al., 2016; Graziano et al., 2019). Since the identification of the Murine Norovirus (MNV) in 2003 (Karst et al., 2003), that can be easily propagated *in vitro* in murine myeloid lineage cells (Wobus et al., 2004) and naturally infect mice, MNV has emerged as a relevant surrogate to study the pathophysiology of Noroviruses.

MNV possesses a linear genome of approximately 7.5 kb (Robilotti et al., 2015). The genome is capped at the 5′ end with the non-structural NS5 protein, which acts as a recruiter of eukaryotic initiation factors for viral genome translation and as a primer for the RNA dependent RNA polymerase (NS7) among other functions (Goodfellow, 2011). The MNV genome has four open reading frames (ORF) that encode a total of nine proteins. ORF1 encodes for a precursor polyprotein that is then proteolytically cleaved into six non-structural proteins (including NS5 and NS7) by NS6 whereas ORF2 and 3 encode for structural viral proteins (VP1, the major capsid protein, and VP2, the minor capsid protein, respectively) and ORF4 (only found in the genomes of MNV) encodes a postulated virulence factor 1 (VF1) (Ludwig-Begall et al., 2021).

Viruses, being obligate intracellular parasites, must modulate hosts in order to highjack their cellular machinery and successfully replicate. Consequently, host cells have developed an arsenal of defence mechanisms against viral infections. MNV is known to mostly trigger innate immune system dependent antiviral responses (Karst et al., 2003; Karst and Wobus, 2015) that are partially regulated by ubiquitination (Davis and Gack, 2015).

Ubiquitination is an ATP-dependent post-translational enzymatic reaction that results in the covalent addition of one or more ubiquitin proteins to the lysine residue of a target substrate. This conjugation enzymatic reaction is accomplished via the cascading actions of three different types of enzymes: the E1 activating enzyme, the E2 conjugating enzyme and the E3 ligases (Pickart and Eddins, 2004). Subsequent ubiquitin molecules can be added to one of the seven lysine residues (K6, K11, K27, K29, K33, K48, K63) or the methionine at the N-terminus of the preceding ubiquitin (Komander and Rape, 2012). The fate of those ubiquitinated proteins is determined by the type of chain produced by polyubiquitination: K48-linked ubiquitin chains are well-known for their involvement in the proteasomal degradation of the ubiquitinated substrate (Davis and Gack, 2015), K63-linked ubiquitin chains have been shown to participate in signal transduction and endocytosis among other functions (Davis and Gack, 2015) and K29-linked ubiquitination was shown to participate in the regulation of many signaling pathways and in some neurodegenerative diseases (Tracz and Bialek, 2021).

Specifically regarding cellular responses to viral infections however, ubiquitination was shown to regulate Stat1 activation to modulate Type-I interferon (IFN) signalling (Zuo et al., 2020). Ubiquitination is also implicated in the stability of pathogen recognition receptors (PRRs) retinoic acid-inducible gene I (RIG-I) and melanoma differentiation-associated protein 5 (MDA5) and more broadly the RIG-I-like Receptors (RLR) pathway, which recognizes cytosolic viral RNA (Davis and Gack, 2015). Indeed, both K48 and K63 linked chains were shown to be heavily involved in the degradation or activation respectively of many effectors involved in this pathway like the mitochondrial antiviral-signalling protein (MAVS) with which RIG-1 and MDA5 interact (Zhang et al., 2018), thereby influencing the activation of IRF3/7, NF-κB and the production of type I IFNs (Thompson et al., 2011; Jensen and Thomsen, 2012). Other innate immune pathways influenced by ubiquitination regulation include the TLR signalling pathway and the cGAS-STING pathway (DNA detection). These in turn activate or repress several downstream proteins related to TNF dependent signalling pathways and hence ultimately regulate NF-κB transcriptional activity leading to multiple levels of anti-viral responses (Davis and Gack, 2015; Hu and Sun, 2016).

The impact of ubiquitination dependent mechanisms in norovirus propagation is poorly understood. On one hand, the replication of several RNA viruses, including the murine norovirus strain MNV-1, was shown to be inhibited when the ubiquitination response was prolonged via specific inhibitors against deubiquitinase (DUB) enzymes resulting in lowered removal of ubiquitin moieties (Gonzalez-Hernandez et al., 2014; Passalacqua et al., 2016). On the other hand, ubiquitination can also promote the anti-noroviral immune responses in the host cell. It was recently shown that amongst other noroviral proteins, the NS3 protein (NTPase) has a conserved recognition motif for binding with the E3-ubiquitin ligase TRIM7 which makes it targetable for TRIM7-dependent ubiquitination and proteasomal degradation. This, in turn, relieves the NTPase-dependent inhibition of IFN-β secretion thus restoring type I IFN anti-viral innate immunity (Liang et al., 2022). Also, in a CRISPR activation screen in HeLa cells expressing the MNV receptor CD300lf, Trim7 was identified as a strong hit that inhibited post-entry MNV replication. The mechanism of this inhibition was further confirmed using a similar type of approach in the BV2 murine microglial cell line. TRIM7 binding with NS6 was found to be dependent on a conserved cleavage site between NS6 and NS7. Mutating this binding site abrogated the interaction between NS6 and TRIM7 and hence the resulting inhibition of viral replication but also favored the emergence of non-NS6/NS7-cleavable low level replicating viral variants (Sullender et al., 2022).

In this study, we have analyzed the impact of ubiquitination on the propagation of the persistent MNoV_S99 strain. We have generated cell lines enriched for the expression of either WT Ubiquitin or Ubiquitin bearing mutations that block the formation of given polyubiquitin chains. This enabled us to identify an unexpected beneficial role for functional K48-dependent polyubiquitin chains formation on MNoV_S99 propagation, while abrogating K29- or K63-Ubiquitins chains had no impact.

## Material and methods

All media and supplementation products were purchased from Thermo Fisher unless otherwise specified.

### Cell culture and virus

The RAW264.7 cell line was purchased from ATCC and grown in DMEM (Dulbecco’s modified Eagle’s medium), supplemented with 10% Fetal bovine serum (Eurobio), 1% Penicillin- Streptomycin and 1% Sodium pyruvate at 37°C with 5% CO2 and 80-90% humidity. The cells were subcultured every 2-3 days at a dilution of 1/5 or 1/10.

The mouse norovirus strain S99 (termed MNoV_S99) (GenBank accession no. DQ911368) was obtained from Prof. P. Maris (ANSES Fougères Laboratory (France)). Viral stocks were produced in RAW264.7 cells.

For infection experiments, cells were seeded at 5×10^5^ to 1×10^6^ cells per well. The next day, just before the infection with MNoV_S99, cells were counted to ensure an adequate determination of a multiplicity of infection (MOI) of 0.1, 1 or 10 depending on the experiment. Cells were infected in a final volume of 1mL cell culture medium. After 2h, 8h or 16h of infection, cells were either fixed with a solution of 4% Paraformaldehyde (PFA) (Sigma-Aldrich) for immunofluorescence experiments, or lysed in RLT buffer (from RNeasy kit, Qiagen) for total RNA extraction for RT-qPCR analysis or total proteins were extracted in PY lysis buffer (Tris-HCL (1M, pH 7.4), NaCl (5M), EDTA (0.5M), 1% Triton X-100 (Sigma-Aldrich)) supplemented with protease and phosphatase inhibitors (Halt™ Protease & Phosphatase Inhibitor Cocktail, Thermo Scientific) for Western Blotting.

### Transfection and selection

RAW264.7 cells were plated in 6 wells plates and transfected (Polyfect^®^, QIAGEN) with 1μg of pEYFP-C3-HA-Ubiquitin expressing either the WT Ubiquitin or constructs bearing point-mutations that changed lysin residues to arginine at the position 29, 48 or 63 (respectively termed K29R, K48R and K63R) (described in (Dupont et al., 2009)). Transfected cells were left at 37°C with 5% CO2 and 80-90% humidity for at least 24h before Fluorescence-activated cell sorting (FACS) using the Yellow fluorescent protein (YFP) signal. YFP+ sorted cells were amplified in the culture medium supplemented with 1000μg/mL of Geneticin (G418) (Gibco). Each culture was further sorted and selected until a sufficiently enriched (at least >50%) YFP+ population for each cell line was obtained. MNoV infection experiments with the cells overexpressing the various YFP-Ubiquitin constructs were performed as described above with the parental RAW264.7 cells.

### Western Blot

Total protein concentration in cleared cell lysates was determined using Pierce™ BCA Protein Assay Kit (Thermo Scientific). Equalized amounts of protein samples were reduced with loading buffer (Laemmli buffer supplemented with Dithiothreitol (DTT) (Sigma Aldrich)) and incubated at 95°C to 100°C for 5 min to 10 min. Proteins were separated according to their molecular weight by SDS-polyacrylamide gel electrophoresis (PAGE) (4-15% or 4%-20% Precast gel, BIO-RAD) and subsequently transferred to a 0.2 µm PVDF membrane (BIO-RAD). The membrane was blocked with a solution of Tris Buffered Saline with 0.1% Tween-20 (Sigma-Aldrich) containing 5% Milk before incubation overnight (ON) at 4°C with specified primary antibody (see **Table 1**). The next day, the membrane was exposed to the appropriate secondary antibody coupled with Horseradish peroxidase (see **Table 1**) for 1h at room temperature (RT). The proteins were revealed with Pierce™ ECL Plus Western Blotting Substrate (Thermo Scientific) and SuperSignal™ West Femto Maximum Sensitivity Substrate (Thermo Scientific) using the FUJIFILM Luminescent Image Analyzer LAS-3000 or the Amersham™ ImageQuant 800. Images were analysed and quantified with Image J software (NCBI).

**Table 1:** Antibodies used for western blotting.

Primary antibodies	Source	Dilution	Manufacturer
anti-polyUbiquitin (starting from 10-12kDa)	Mouse	1/1000	Enzo #BML-PW8805
anti-NS7(60kDa)	Rabbit	1/4000	Self-produced
anti-pIκBα (40kDa)	Mouse	1/1000	Cell Signaling Technology #9246
anti- IκBα (39kDa)	Rabbit	1/1000	Cell Signaling Technology #4812
anti-β Actin (45kDa)	Rabbit	1/1000	Cell Signaling Technology #4967
anti-β Tubulin (55kDa)	Mouse	1/5000	Sigma-Aldrich #T5201

Secondary antibodies	Source	Dilution	Manufacturer
anti-rabbit_HRP	Goat	1/5000	Jackson Laboratories #711-035-152
anti-mouse_HRP	Goat	1/5000	Jackson Laboratories #115-005-003

Mean intensities of the bands of interest were normalized with the β-actin or β-tubulin intensities from the same lane and normalized with the control non-infected condition. Averaged values from at least three independent experiments were compared between different time-points, doses of infections or cell lines.

### Immunofluorescence assay

Fixed cells were permeabilized in blocking solution (PBS with 30% FBS) supplemented with 0.1% Triton-X100 (Sigma-Alrich), saturated with the blocking solution and incubated ON at +4°C with specified primary antibodies (**Table 2**) in blocking buffer. The next day, the cells were incubated 1h at RT with the secondary antibodies conjugated with fluorophores (**Table 2**) and nuclei were stained with DAPI (Cell Signaling Technology). The VP1 antibody was kindly gifted to us by Dr Christiane Wobus (University of Michigan, USA) see (Kolawole et al., 2014).

**Table 2:** Antibodies used for immunofluorescence experiments.

Primary antibodies	Source	Dilution	Manufacturer
anti-polyUbiquitin	Mouse	1/100	Enzo #BML-PW8805
anti-NS5	Rabbit	1/600	Self-produced
anti-Nf-κB	Rabbit	1/1000	Cell Signaling Technology #8242
anti-dsRNA	Mouse	1/1000	CliniSciences #RNT-SCI-10010200
anti-Vp1	Mouse	1/100	Clone 5C47 (C.E. Wobus, University of Michigan, USA)

Secondary antibodies	Source	Dilution	Manufacturer
anti-mouse_Alexa-555	Goat	1/1000	Invitrogen #A21422
anti-mouse_Alexa-633	Goat	1/1000	Invitrogen #A21050
anti-rabbit_Alexa-555	Goat	1/1000	Invitrogen #A32732
anti-rabbit_Alexa-647	Donkey	1/1000	Invitrogen #A31573

Cells were imaged with the spinning disk confocal microscopy (Nikon CSW1) using the super resolution module (Live SR Gataca) with a 40x or 60x objective. Images were analysed and quantified with Image J software (NCBI) or Imaris (Oxford Instruments).

### Image quantification

Using Image J, 3D-stacks of images were Z projected (sum of maximum intensities). Projected images had the stained marker of interest’s signal area thresholded and normalized with the signal corresponding to total cell surface area for each image. 3 to 5 images (> 50 cells) per condition and per experiment were analysed. Each experiment was repeated at least 3 times.

For the NF-κB nuclear translocation analysis, images were analyzed with Imaris. The “Cell” module enabled the detection of the nucleus (thresholding of the DAPI fluorescence channel) as well as the cytoplasm (thresholding of the YFP fluorescence channel) and the marker of interest NF-κB p65 signal for each cell. From the statistics table, the sum intensity of voxel fluorescence constituting the nucleus as well as the cytoplasm were obtained. The calculated ratio: r = Sum Intensity Nucleus/(Sum Intensity Nucleus + Sum Intensity Cytoplasm) was then used as a measure of the NF-κB p65 signal corresponding to the localization in the nucleus area.

### RT-qPCR

Total RNA extraction was performed using the RNeasy Mini Kit (Qiagen). Viral genome levels were determined by RT-qPCR using the Takyon™Dry No Rox One-Step RT Probe Mastermix (Eurogentec) as previously described in (Muharram et al., 2023).

For cellular gene expression measurement, total extracted RNA was reverse-transcribed with a High-Capacity cDNA Archive Kit (Applied Biosystems). Gene expression was quantified by cDNA amplification in a QuantStudio3 real-time PCR system (Applied Biosystems) using Low ROX SYBR MasterMix (Eurogentec). Relative mRNA levels (2^-ΔΔCt^) were determined by comparing the PCR cycle thresholds (Ct) for the gene of interest and the housekeeping genes Ac*tb, B2m, Gapdh* (ΔCt) and the ΔCt values for the infected and control cells (ΔΔCt). The Ct threshold was set to 35 cycles. Sequence of primers has been described previously in (Mondemé et al., 2024) or are available upon request.

### Titration

The total amount of infectious MNoV_S99 virions in each condition was determined using the TCID50 titration method as described in (Hwang et al., 2014) from the supernatants of infected cells for 24 h.

### TNF ELISA

The concentration of murine TNF was determined from the supernatants (n = 3) of mock-treated or virus-infected cells for 24 h using the mouse TNF ELISA kit (R&D Systems) according to manufacturer instructions.

### Statistical analysis

All the data were processed for graphical representation and analyzed for statistical analysis with GraphPad Prism 10 software (see figure legends for details about used tests).

## Results

### MNoV_S99 infection triggers upregulation of the ubiquitination response

**Figure 1:**
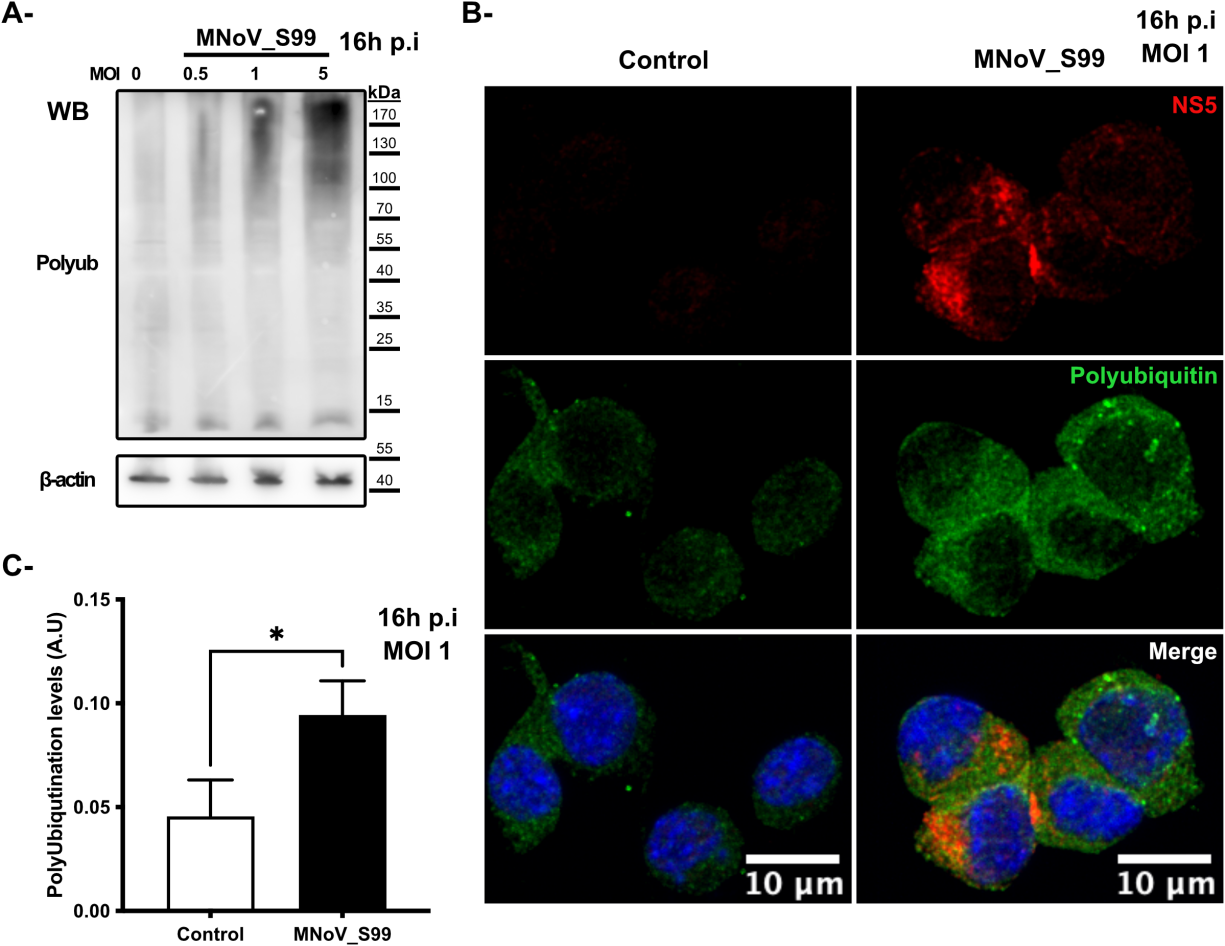
Ubiquitination response modification following MNoV_S99 infection. **(A)** RAW264.7 cells were infected with MNoV-S99 (MOI 0.5, 1, 5 or mock infected (0) for 16h). Lysates were subjected to a Western Blot detection using antibodies against polyubiquitin and ß-actin. **(B)** RAW264.7 cells were infected with MnoV_S99 (MOI 1, 16h), or mock treated (Control). Fixed and labelled cells were imaged using confocal spinning-disk microscope with a 40x objective. Viral protein NS5 is displayed in red (NS5) and the polyubiquitin chains in green (Polyubiquitin). The nuclei are counterstained with DAPI shown in blue in the merged images. **(C)** The area of the polyubiquitination signal was quantified using FIJI/ImageJ and was normalized according to total cell surface, 3 images per condition were used with at least 20 cells per images. Mean ± SD from n = 3 independent experiments are represented, statistical significance was determined with Mann-Whitney test, *p= 0.04.

To initially assess the degree of ubiquitination that occurs during MNV infection, RAW264.7 macrophages were infected with the MNoV_S99 strain at increasing multiplicities of infection (MOI) of 0.5, 1 or 5 for 16h, and the total levels of cellular polyubiquitination were analysed by western blotting (**Figure 1A**). The MOIs and timings of infection were specifically chosen to be physiologically relevant and also empirically determined to provide enough biological material for robust analysis of samples without being too deleterious for the cells. As is typically observed when blotting for ubiquitin or polyubiquitin, a smear can be detected in all conditions which corresponds to the polyubiquitin chains of various lengths and polyubiquitinated proteins at different molecular weights. We can observe, however, an increasing level of smear intensity that follows the increasing MOI (**Figure 1A**) suggesting that ubiquitination levels are correlated with infection levels. Similarly, the detection of polyubiquitin chains by immunofluorescence via confocal microscopy (as seen in **Figure 1B**) shows an increase in the signal corresponding to polyubiquitin (shown in green) in the infected RAW264.7 cells (MNoV_S99 highlighted in red) as opposed to the uninfected RAW264.7 cells. Indeed, subsequent image quantification also revealed a significant increase of about 50% of the polyubiquitin signal in the infected conditions (MOI 1, 16h post-infection (p.i)) versus the mock infected cells (Control) (**Figure 1C**). Overall, these results indicate that the levels of polyubiquitination increase following MNoV_S99 infection.

To further investigate the role of polyubiquitination in response to MNoV infections we transfected RAW264.7 cells with YFP-Ubiquitin_WT, YFP-Ubiquitin_K29R, YFP-Ubiquitin _K48R or YFP-Ubiquitin_K63R constructs (all non-WT constructs encode a ubiquitin fusion protein with the indicated lysine residue being substituted with an arginine thus preventing the formation of the relevant polyubiquitin chain (Dupont et al., 2009)). Additionally, the Yellow fluorescent protein (YFP) fusion protein facilitates the immunofluorescent detection of these constructs when using confocal fluorescence microscopy and YFP+ cell sorting via FACS (see below). Moreover, since the transfected cells express the Neomycin resistance gene cassette, we were able to obtain highly enriched YFP+ cells populations using Geneticin (1000μg/mL) selection and successive rounds of FACS every 2 to 3 passages.

**Figure 2:**
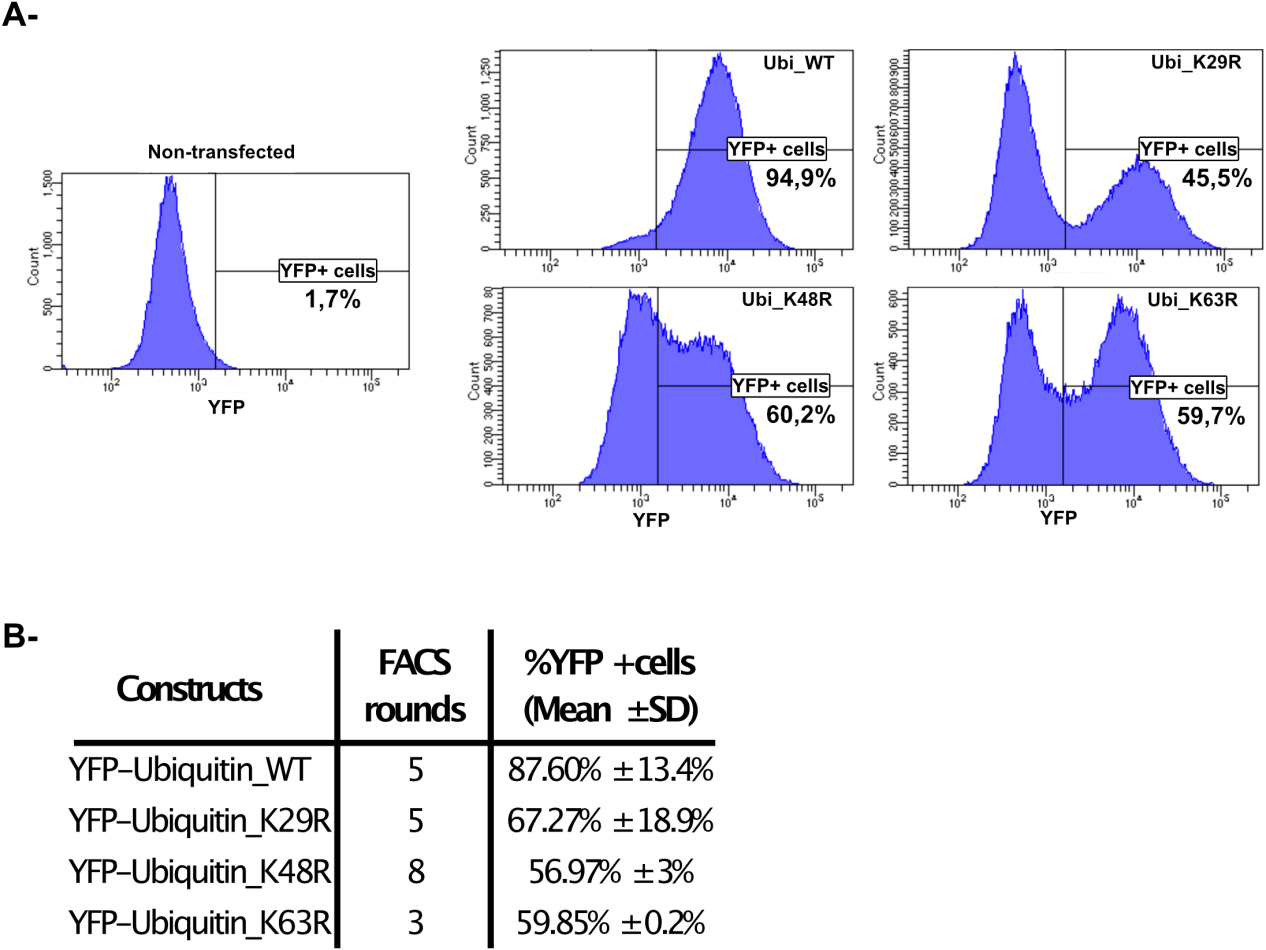
Generation of RAW264.7-YFP-Ubiquitin_WT, _K29R, K48R or K63R cells. **(A)** A representative analysis of the selection and gating performed in the FACS used in the quantification and sorting of YFP positive cells. **(B)** The table shows the number of sorting over the time of selection for each cell line and the obtained % YFP+ cells populations analysed by flow cytometry (mean ± SD, n=3, at least 1x10^5^ events analysed/cell line/measure).

The level of YFP signal for each cell-line was measured by repeated (n = 3) cytometry analysis (**Figure 2A**) at different time points during our cycle of selection. The percentage of stabilized YFP+ cells we obtained following this protocol were: 87.60% ± 13.4% for YFP-Ubiquitin_WT cells, 67.27% ± 18.9% for YFP-Ubiquitin_K29R cells, 56.97% ± 3% for YFP-Ubiquitin_K48R cells and 59.85% ± 0.2% for YFP-Ubiquitin_K63R cells (**Figure 2B**).

### Blocking K48-dependent polyubiquitin chains formation alters MNoV_S99 propagation

**Figure 3:**
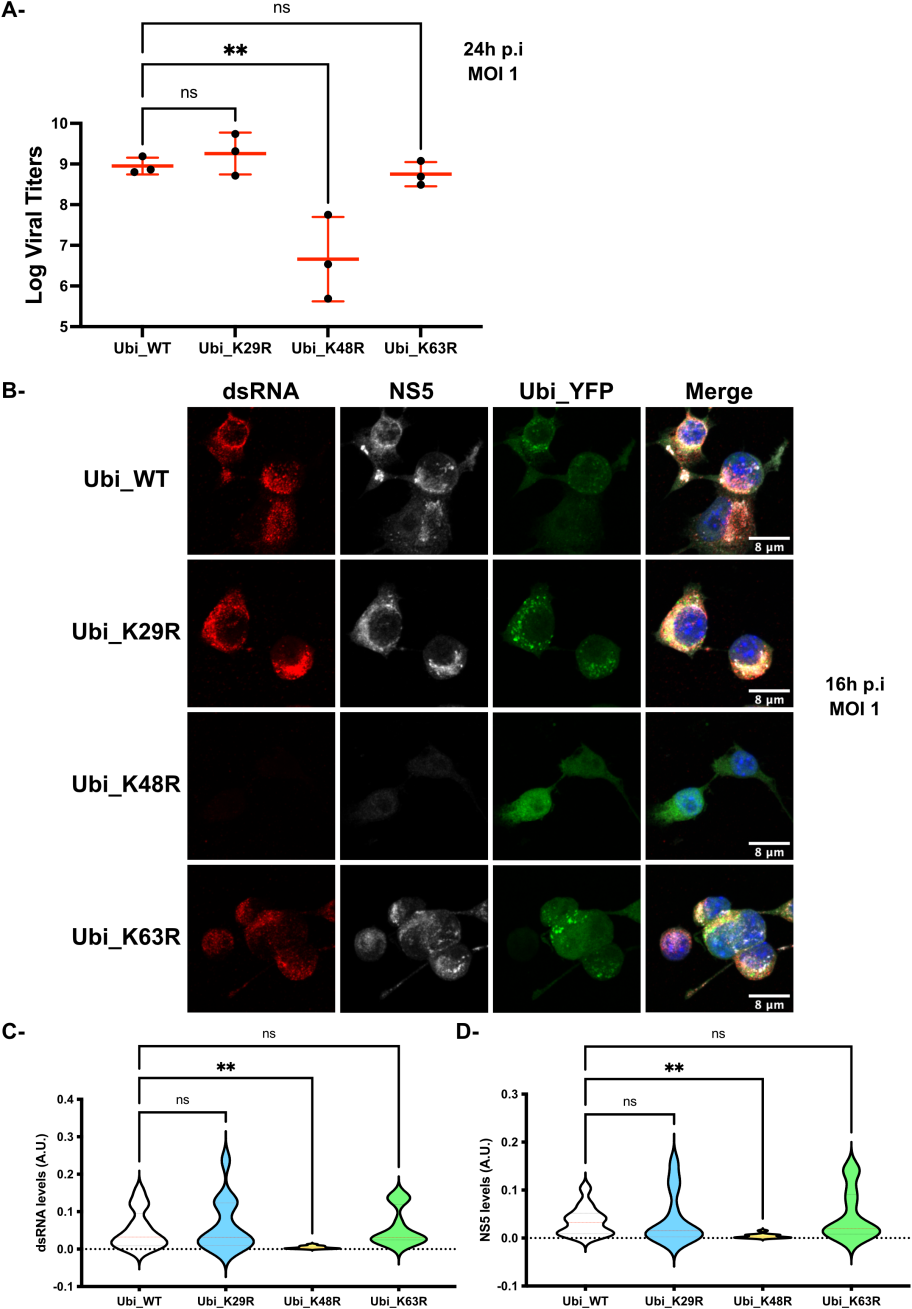
MNoV_S99 propagation is impaired in Ubi_K48R cells. **(A)** The different cell lines were infected with MNoV_S99 (MOI 1, 24h). Viral titers were determined from supernatants using the TCID_50_ titration method (mean ± SD, n=3). Statistical differences were determined with one-way ANOVA test, ** p= 0,0044. **(B)** RAW264.7-YFP-Ubiquitin_WT, K29R, K48R- and K63R transfected cells (termed Ubi_WT, Ubi_K29R, Ubi_K48R and Ubi_K63R respectively) were exposed to MNoV_S99 (MOI 1, 16h). Fixed cells were marked with anti-dsRNA antibodies (shown in red) and anti-NS5 antibodies (in grey), the nuclei were counterstained with DAPI (blue). Cells were imaged by spinning-disk confocal microscopy with a 40x objective. **(C)** Total cell areas of the viral dsRNA and **(D)** NS5 expression were quantified using FIJI/ImageJ and was normalized according to total cell surface. 4-5 images were used per condition with at least 20 cells per images. Mean ± SD from n = 3 independent experiments are represented, statistical significance was determined with ANOVA test, **p<0.005; ns indicates non-significant differences.

To examine the consequences of polyubiquitin on MNoV replication, RAW264.7_YFP-Ubiquitin_WT, _K29R, _K48R or _K63R transfected cells (subsequently abbreviated as Ubi_WT, Ubi_K29R, Ubi_K48R and Ubi_K63R respectively) were infected with MNoV_S99 (MOI 1, 24h) and production of the infectious virus was determined. At 24h p.i, infected supernatants from the different cell-lines were harvested and MNoV titers were determined by the TCID50 titration method (**Figure 3A**). Interestingly, in comparison with the cells overexpressing the WT Ubiquitin construct, which can generate all chain types, neither the reduced K29-dependent nor K63-dependent polyubiquitin chain formation showed any significant difference in infectious virus production. However, the supernatants from Ubi_K48R cells showed a massive and statistically significant reduction in viral titers of around 3 logs. Similar results were obtained when we compared the production of MNoV_S99 in Ubi_K48R cells versus the parental non-transfected RAW264.7 cells (**Supplementary Figure 1A**). These results indicated a crucial role for K48-dependent polyubiquitin chain formation in the production of infectious MNoV_S99.

To determine if K48-dependent polyubiquitin chain formation was required for virion formation only or for some earlier stages of the virus life cycle, we analysed the presence of dsRNA molecules, indicative of the intermediate state in single stranded RNA virus genome replication, and the expression of the viral non-structural proteins NS5 in the Ubi_WT, Ubi_K29R, Ubi_K48R and Ubi_K63R cell lines infected with MNoV_S99 (MOI 1, 16h) by immunofluorescence confocal microscopy. Consistent with the viral titration data, only the MNoV_S99 infected Ubi_K48R cells showed decreased levels of NS5 and dsRNA (**Figure 3B**) (equivalent full-size images for dsRNSA are shown in **Supplementary Figure 2**), when compared to the Ubi_WT, Ubi_K29R and Ubi_K63R cell lines. Similar results were obtained when comparing Ubi_K48R with non-transfected cells (**Supplementary Figure 1B**). The image quantifications from several independent infection experiments (n = 3) confirmed the statistically potent reduction in both dsRNA and NS5 levels (**Figures 3C, D** respectively) in Ubi_K48R cells in comparison with Ubi_WT cells. No such effects were measured from Ubi_K29R or Ubi_K63R cells.

Altogether, our results indicate that the MNoV_S99 viral lifecycle strongly relies on the capacity of the host cell to generate K48-dependent polyubiquitin chains.

### Impaired production of MNoV_S99 in Ubi_K48R cells is not caused by compromised viral entry

To further study the mechanisms that could explain the need for the presence of K48-dependent polyubiquitin chains in the propagation of MNoV_S99, we conducted additional experiments only comparing the differences between Ubi_K48R and Ubi_WT cells. We refined our analyses to focus on the different steps of the virus life cycle including virus entry, genome amplification, viral production and infectious virus release.

**Figure 4:**
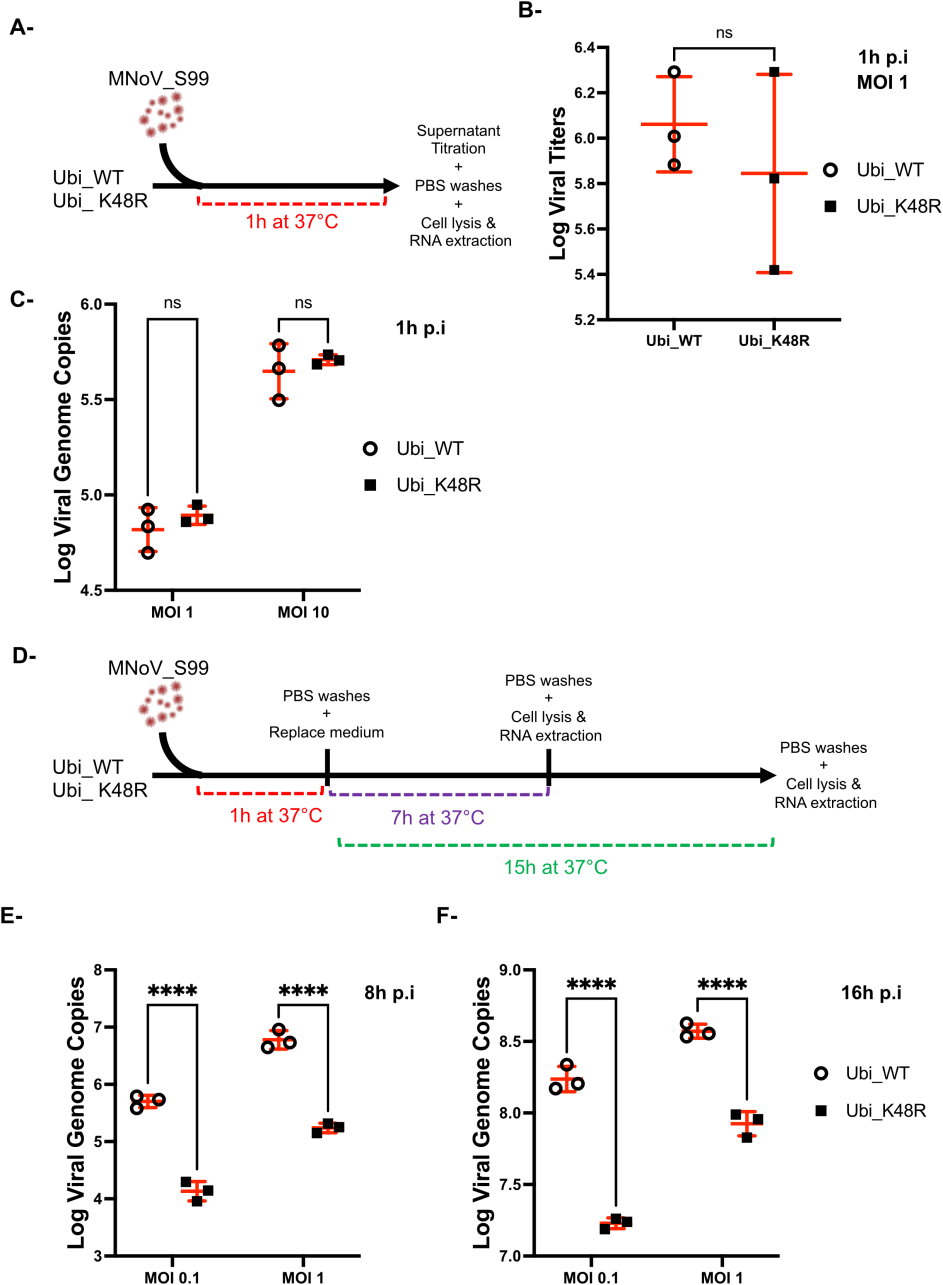
MNoV_S99 entrance is not compromised in Ubi_K48R cells. **(A)** Schematic representation of the experimental plan used to measure the viral entrance in Ubi_WT and Ubi_K48R cells infected with MNoV_S99 at MOI 1 for 1h, at 37°C. **(B)** Residual non-internalised viruses’ amounts were compared using TCID_50_ titration method (mean ± sd, n = 3). **(C)** Viral genome copies were quantified with RT-qPCR analysis from Ubi_WT or Ubi_K48 cells infected with MNoV_S99 MOI 1 or 10 for 1h, at 37°C. **(D)** Schematic representation of the experimental plan used to measure viral genome copies from Ubi_WT or Ubi K48 cells at MOI of 0.1 or 1 for a total of 8h or 16h of infection with MNoV_S99. The respective measures are shown in **(E, F)** as total number of MNoV genome copies (mean ± SD) from n = 3 independent experiments. Statistical significances were determined with 2-ways ANOVA test, ****p<0.0001; ns indicates non-significant differences.

To more carefully examine if viral attachment and entry were impaired in cells expressing the mutated (Ubi_K48R cells) versus the WT ubiquitin form (Ubi_WT cells) both cell types were exposed to MNoV_S99 (MOI 1) for 1h at 37°C. The remaining supernatants were then recovered at 1h p.i and analysed by the TCID50 titration method (as seen in **Figure 4A**). There was no observable difference in the number of infectious viruses remaining in the supernatants from the Ubi_WT or Ubi_K48R cells (**Figure 4B**). This suggests that the infectivity of MNoV_S99 into the Ubi_K48R cells is not altered or attenuated. Similarly, no quantitative differences in MNoV_S99 genome copies in the infected cells could be observed when RT-qPCR analyses were performed on total RNA extracted from both cell types exposed to MNoV_S99 (MOI 1 or 10) for 1h at 37°C (**Figure 4C**).

Taken together these data indicate that the impact of polyubiquitination we previously observed on MNoV_S99 production in Ubi_K48R cells is not due to restricted attachment or entry and thus must be associated with defects in later stages of the MNoV cycle.

Classically, in RAW264.7 cells, the replication of MNoV is effectively measurable starting at 8h post-infection (Levenson et al., 2018). In our experiments, we have measured the number of MNoV_S99 genome copies at 8h and 16h post-infection by RT-qPCR in Ubi_WT or Ubi_K48R cells infected at MOI of 0.1 or 1 (as seen in **Figure 4D**). At both time points, a significantly decreased number of genome copies can be measured in Ubi_K48R cells compared to Ubi_WT cells with both multiplicity of infection (**Figures 4E, F**).

**Figure 5:**
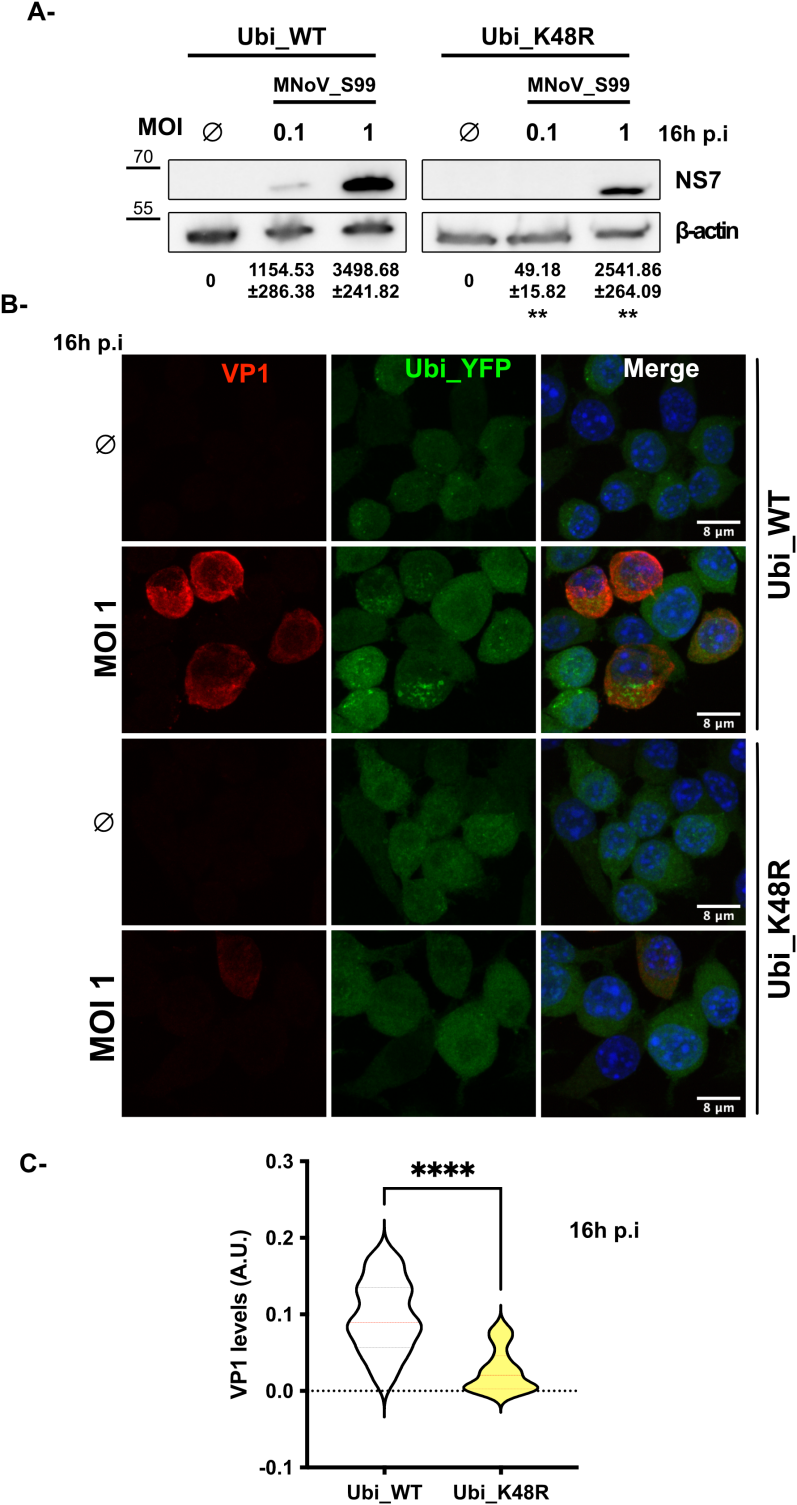
MNoV_S99 lifecycle is impaired in Ubi_K48R cells. **(A)** Ubi_WT and Ubi_K48R cells were infected with MNoV_S99 (MOI 0.1, 1 or mock infected (0) for 16h). Total proteins lysates were subjected to WB. The viral marker NS7 band intensities were measured and normalized with ß-actin (mean ± sd, n = 3). The variations of NS7’s levels were compared at both MOIs between the two cell types using Student’s T.test (**p = 0.0013 and **p= 0.0049 respectively at MOI 0.1 and MOI 1). **(B)** MNoV_S99 infected (MOI 1, 16h) Ubi_WT or Ubi_K48R fixed cells were stained for the late viral marker VP1 (in red). The YFP_Ubiquitin signal is shown in green. The nuclei were counterstained with DAPI (blue). Cells were imaged by spinning-disk confocal microscopy with a 60x objective. **(C)** The area of the viral VP1 signal was quantified using FIJI/ImageJ and was normalized according to total cell surface (5 images/condition and at least 30 cells per image from n = 3 independent experiments were analyzed). Statistical significance was determined with Mann-Whitney test ****p<0.0001.

Consistently, the defect in the early steps of the MNoV_S99 lifecycle can also be seen when comparing the level of expression of the non-structural viral protein NS7, the virus-derived RNA-dependent RNA polymerase by western blot. Indeed, MNoV_S99 infected Ubi_WT cells showed increasing levels of NS7 in a dose-dependent manner (**Figure 5A**, left panels). However, a reduced production of NS7 can be observed in the infected Ubi_K48R cells, even at the highest dose of infection (MOI 1) (**Figure 5A**, right panels) as suggested by the quantification of the NS7 signal normalised with β-actin levels.

Next, we used IF to analyze how the expression of the major viral capsid protein VP1, which can be considered a relatively late marker of the MNoV replication cycle, was affected by the reduction in formation of K48-dependent polyubiquitin chains in MNoV_S99 infected (MOI 1, 16h p.i) cells. In agreement with our previous observations on the production of dsRNA and NS5 (**Figures 3B–D**) or NS7 (**Figure 5A**), we observed that the expression of VP1 was drastically and significantly reduced in the MNoV_S99 infected Ubi_K48R cells in comparison with the Ubi_WT infected cells (**Figures 5B, C**).

Taken together, our data strongly point toward defects in multiple stages of the viral lifecycle rather than a restriction at the level of attachment and/or entry, in the cells deficient in producing K48-dependent polyubiquitin chains.

### TNF dependent pro-inflammatory signalling is upregulated in Ubi_K48R cells

MNoV is known to trigger and activate mostly innate antiviral immune responses (Ludwig-Begall et al., 2021), hence, we focused on analysing how the molecular signalling cascades related to innate immunity are disturbed by the alterations caused to the formation of K48 dependant polyubiquitin chains in our Ubi_K48R mutant cells.

**Figure 6:**
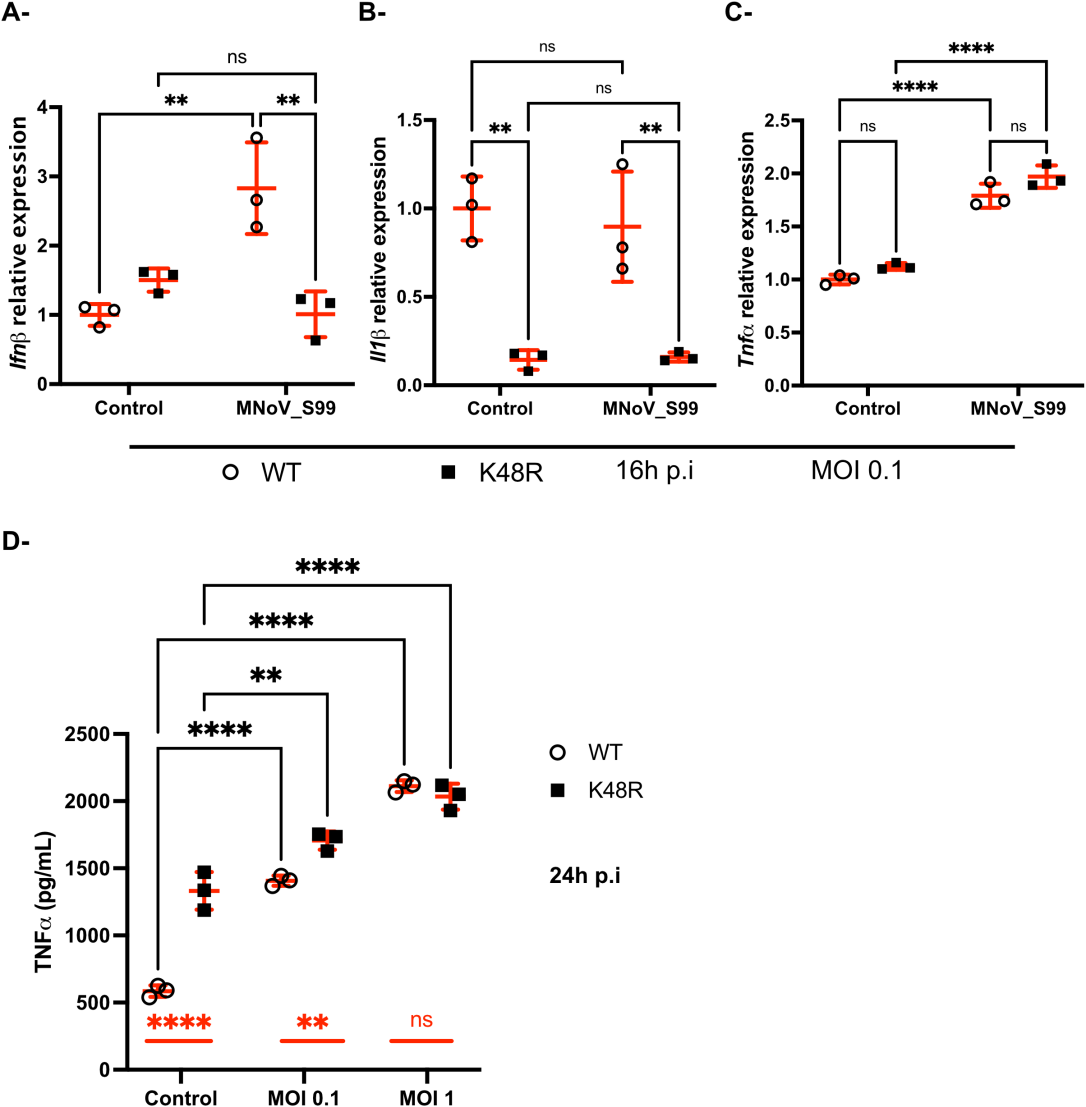
Differences in the regulation of anti-viral immunity against MNoV_S99 in Ubi_WT versus Ubi_K48R cells. Ubi_WT or _K48 cells mock infected (Control) or infected with MNoV_S99 (MOI 0.1, 16h) were analyzed by RT-qPCR for expression of *Ifnb* in **(A)**, *Il1b* in **(B)** and *Tnf* in **(C)** relatively to housekeeping genes. Statistical significances were determined with 2-ways ANOVA test, **p=0.002 and ****p<0.0001. **(D)** Quantification by ELISA of secreted TNF in the supernatants from Ubi_WT or _K48R cells infected with MNoV_S99 (MOI 1, 24h) or mock infected (Control). Mean ± sd are shown from n = 3 independent experiments, statistical significances were determined with 2-ways ANOVA test, **p=0.002 and ****p<0.0001; ns indicates non-significant differences. The differences between non-infected and infected cells are shown with bars in black for both cell lines. The differences between Ubi_WT and Ubi_K48R cells are shown with red bars.

Thus, Ubi_WT and Ubi_K48R cells were infected with MNoV_S99 at an MOI of 0.1 for 16h and RT-qPCR analyses were performed on extracted RNA samples. We measured the relative mRNA transcripts levels of multiple cytokines generally implicated in inflammation, namely IFNβ, IL1β and TNF in order to decipher any potential differences in the way both cell lines react to MNoV_S99 infection. Interestingly, while Ubi_WT cells were shown to have a statistically significant threefold increase in *Ifnb* mRNA levels following infection with MNoV_S99, Ubi_K48R cells appeared unresponsive (**Figure 6A**). Additionally, the MNoV_S99 infection did not significantly alter the transcription of *Il1b* (**Figure 6B**). However, *Il1b* transcription already appears to be significantly reduced at the basal level in Ubi_K48R mock infected (Control) cells (**Figure 6B**).

Conversely, increased transcription of the *Tnf* gene was measured following MNoV_S99 infection in both Ubi_WT and Ubi_K48 cells (**Figure 6C**). This was further confirmed, when secreted levels of TNF were analysed by ELISA from the supernatants of both cell lines infected for 24h at an MOI of 0.1 or 1. TNF levels were significantly increased in comparison with mock treated cells (Control) (**Figure 6D**) for both cell lines.

Surprisingly, a significant increase was also already detectable when comparing the TNF levels in the supernatants of the uninfected Ubi_K48R cells versus the uninfected Ubi_WT cells (**Figure 6D**). The difference between the two cell lines seemed to decrease with increasing doses of viral infections.

This observation suggests that the TNF signalling pathway is probably regulated at the protein level in the Ubi_K48R cells. Indeed, impaired proteasomal degradation is expected in cells that cannot form K48-dependent polyubiquitin chains.

Thus, the baseline increased secretion of TNF could perhaps partially explain the resistance the Ubi_K48R cell line shows upon infection with the MNoV_S99.

### The deficiency of K48-dependent polyubiquitin chains production is associated with an excessive activation of the NF-κB pathway

To further characterise the effect of the heightened secretion of TNF observed within the Ubi_K48R cell line, the potential downstream effects of TNF were investigated. We focused on the NF-κB pathway that is well-known to be induced following TNF activation (Wajant et al., 2003).

**Figure 7:**
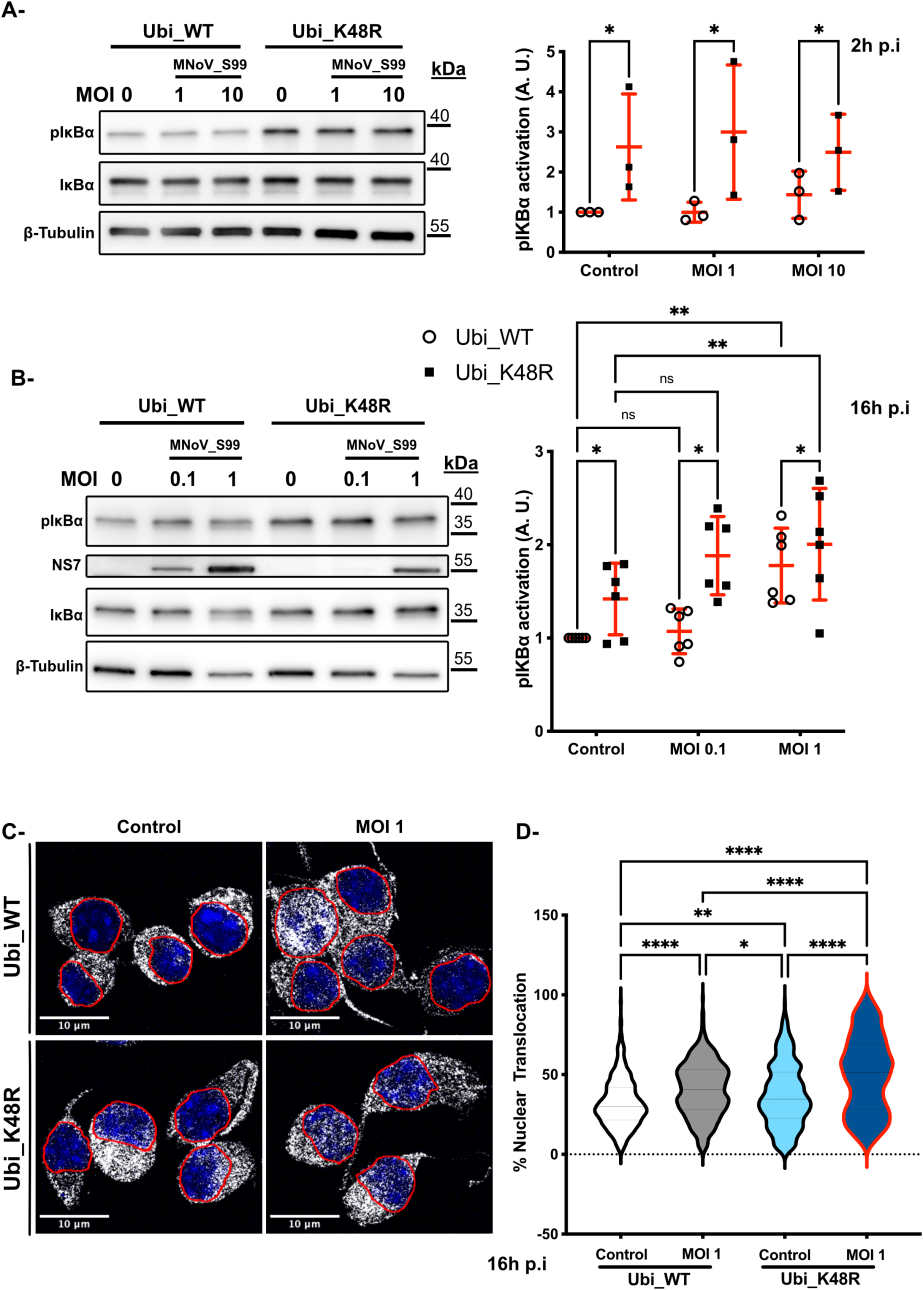
Upregulation of NF-κB activation in Ubi_K48R cells. **(A)** Total proteins lysates from Ubi_WT and Ubi_K48R cells infected for 2h with MNoV_S99 (MOI 1, 10 or mock infected (0)) or were subjected to a Western Blot analysis (left panel). Quantification (mean ± sem, n = 3) of protein expression levels were compared after normalization with ß -Tubulin expression and relative to pIκBα levels in the control Ubi_WT mock infected condition (right panel). Statistical differences were analyzed using 2-ways ANOVA test *p=0.03. **(B)** Comparisons of the indicated proteins levels by WB (left panel) in cells lysates from 16h infected cells (MOI 0.1 or 1) and quantifications (mean ± sem, n = 6) of pIκBα levels normalized with ß -Tubulin expression and relative to pIκBα levels in the control Ubi_WT mock infected condition are presented in the right panel. Statistical differences were analysed using 2-ways ANOVA test *p=0.01, **p=0.002, ****p<0.0001; ns indicates non-significant differences. **(C)** MNoV_S99 infected (MOI 1, 16h) Ubi_WT or _K48R fixed cells were stained with anti-Nf-κB antibodies (in gray). The nuclei were counterstained with DAPI (blue). Cells were imaged by spinning-disk confocal microscopy with a 60x objective. **(D)** Quantification of the percentages of Nf-κB nuclear translocation were measured with Imaris (5 images/condition and at least 20 cells per image from n=3 independent experiments were analyzed). Statistical significance was determined with one-way ANOVA test *p=0.04, **p=0.005, ****p<0.0001.

Initially, western blot analyses were performed on MNoV_S99 infected Ubi_WT and Ubi_K48R cells to determine the levels of phosphorylated IκBα (pIκBα) (**Figure 7A**, left panels). After 2h of exposure to MNoV_S99 at an MOI of 1 or 10, a statistically significant increase in pIκBα levels was observed in Ubi_K48R cells as opposed to Ubi_WT cells in all conditions (**Figure 7A**, right panel). However, as shown previously with the secretion of TNF (**Figure 6D**), the increased levels of pIκBα were also detectable at the basal level even before MNoV_S99 infection in Ubi_K48R cells.

Subsequently, at 16h p.i at an MOI of 0.1 or 1 a significant increase in pIκBα expression can be seen for both cell lines following MNoV_S99 infection, but only at the highest multiplicity of infection (**Figure 7B**). Again, the basal level excessive activation of pIκBα was still detectable in the Ubi_K48R cells as compared to Ubi_WT cells. Although, after 16h of infection, the Ubi_WT cells pIκBα levels appeared to increase similarly to that in the Ubi_K48R cells. Similar results were obtained when Ubi_48R cells were compared with non-transfected cells (**Supplementary Figure 1C**).

Finally, to confirm whether the higher levels of pIκBα induced a higher degree of NF-κB p65 nuclear translocation, we analysed the localisation of NF-κB p65 by immunofluorescence confocal microscopy in both cell lines before and after infection with MNoV_S99 (MOI 1) (**Figure 7C**). As expected, our observed activation of both TNF and pIκBα in the MNoV-S99 infected Ubi_K48R cells, resulted in an increased nuclear translocation of NF-κB p65 (**Figure 7D**). Of note is that nuclear localisation of NF-κB p65 was significantly increased at the basal level but further increased in the infected cells. This is in contrast to Ubi_WT cells, where the nuclear translocation of NF-κB p65 was only detected in MNoV_S99 infected cells.

Taken together, these results seem to suggest that the deficiency in K48-dependent polyubiquitin chains production induces a higher TNF basal secretion that results in increased IκBα phosphorylation which in turn provokes an increased NF-κB nuclear translocation in Ubi_K48R cells. The priming effect with the excessive activation of this signalling pathway might explain the resistance to the viral propagation following MNoV_S99 infection in the Ubi_K48R cells.

## Discussion

Considering the vast implication of ubiquitination in many aspects of cellular defence, the fact that viruses have adapted to highjack this versatile system is to be expected (Gustin et al., 2011; Valerdi et al., 2021). Indeed, many steps in the lifecycle of different viruses have been shown to be impacted by ubiquitination. Many viruses (Valerdi et al., 2021), like the Japanese encephalitis virus (Wang et al., 2016), are reported to be negatively impacted by the use of proteasome inhibitors. Additionally, the uncoating of the dengue virus genome was found to be hampered when ubiquitination was inhibited (Byk et al., 2016), and it was shown that the entry of the virus in infected cells was partially influenced by receptor ubiquitination (Dejarnac et al., 2018). Recently, ubiquitination was also demonstrated to directly impact Zika virus pathogenicity as K63-dependent ubiquitination of the viral envelope protein facilitates virus entry in the surrounding cells (Giraldo et al., 2020). Viruses have also been shown to use ubiquitination to enhance the replication of their genome. For example, TRIM6 ubiquitinates the VP35 protein of the Ebola virus thereby enhancing its replication (Bharaj et al., 2017). Other impacted stages are: viral assembly (Influenza A virus protein M2 was shown to have a reduced efficiency in viral assembly when not able to be ubiquitinated) and viral evasion of the cell immune system (by inhibiting IFN production) (Valerdi et al., 2021; Park et al., 2022).

Ubiquitination dependent mechanisms involved in norovirus propagation are as of yet still poorly characterized. In this study, we have analyzed how the MNoV_S99 strain is impacted in RAW264.7 cells where the formation of K29-, K48- or K63-linked polyubiquitination chains formation is hampered in comparison with cells enriched for the WT-Ubiquitin construct that can generate all types of chains. This enabled us to identify an unexpected beneficial role for functional K48-dependent polyubiquitination on MNoV_S99’s propagation, while abrogating K29- or K63-Ubiquitins chains had no such impact. Indeed, Ubi_K48R cells showed decreased expression of several viral markers: the non-structural proteins NS5 and NS7, encoded by ORF1, that are expressed early in the viral cycle; dsRNA molecules, an intermediate in viral replication and the viral main capsid protein VP1, a relatively late marker. Additionally, the number of genome copies of MNoV_S99 was significantly decreased in the infected Ubi_K48R cells compared to Ubi_WT cells. Accordingly, the titers of the infectious virions released in the supernatant of infected Ubi_K48R cells were also severely reduced.

Interestingly, when attempting to decipher at what point within in the viral lifecycle the impact of impaired K48 dependent polyubiquitination is felt the most, we observed that viral attachment and entry does not appear to be perturbed. These findings led us to hypothesize that it is the immunity regulatory aspects of K48-polyubiquitination that play a role in modulating viral replication. Curiously, when looking at the gene expression level by RT-qPCR analysis, we observed a decreased expression in *Ifnb* (a potent anti-viral regulator) and the pro-inflammatory cytokine *Il1b*. This could be related to a strain effect. Indeed, contrary to the widely used acute MNV 1 strain, we, in primary macrophages (Muharram et al., 2023), and others, in RAW264.7 cell lines (Niendorf et al., 2016), have shown that the persistent MNoV_S99 strain triggers a mild to absent interferon response following infection. However, in the previously mentioned study (Muharram et al., 2023) we did measure a pro-inflammatory response in primary macrophages in the form of a *Nod2*-dependent TNF activation post-MNoV_S99 infection.

Accordingly, in this study the *Tnf* gene expression levels were increased in both Ubi_WT and Ubi_K48R MNoV_S99 infected cells in comparison to mock infected cells. Similarly, TNF secretion (ELISA) was also augmented in a dose-dependent manner following MNoV_S99 in both cell lines.

However, we found that the Ubi_K48R cells already had a higher baseline (prior to viral infection) secretion of this cytokine. This was not detected when looking at *Tnf* expression at the mRNA level. This observation potentially highlights a regulation of the TNF signalling pathway at the protein level. Indeed, decreased proteasomal degradation of cytosolic proteins is expected when K48-dependent polyubiquitin chains are hampered.

Similarly, when examining the modulation of the signalling pathway downstream of TNF, we also measured an increased phosphorylation of IκBα and the subsequent NF-κB translocation in Ubi_K48R cells even at a steady state preceding infection with MNoV_S99 but also subsequent to infection. Hence, our data suggests that blocking K48-dependent polyubiquitination tempers the production and release of TNF at the basal level prior to any MNoV_S99 infection. The K48R ubiquitin mutant appears to prime this pro-inflammatory pathway promoting TNF hypersecretion creating a non-permissive environment for MNoV_S99 propagation. However, more work is required to be able to definitively conclude on the matter. Additionally, the ubiquitination process can finetune many host cell defence mechanisms following pathogenic infections. The downstream effectors of pathogen recognition receptors (PRRs), such as MAVS, were shown to be impacted by K48-polyubiquitination (Davis and Gack, 2015), resulting in altered activation of IRF3/7 and NF-κB (Thompson et al., 2011; Jensen and Thomsen, 2012). RIG-I and MDA5 stability are also known to be regulated by ubiquitination (Davis and Gack, 2015) and MDA5 is proven to be an essential player in the anti-noroviral response (McCartney et al., 2008). Similarly, the cGAS-STING pathway, which was recently shown to be involved in the cellular response to MNV (Jahun et al., 2023), is also modulated by ubiquitination (Davis and Gack, 2015; Hu and Sun, 2016).

In conclusion, the data obtained here in our cellular model where K48-dependent ubiquitination is limited hint toward a constitutive alteration of the TNF - NF-κB antiviral signalling cascade. Most likely, the Ubiquitin_K48R mutant negatively influences proteasomal degradation of one or many, yet to be identified, regulatory molecules yielding an upregulation of the activation of TNF - NF-κB signalling pathway, thus creating a norovirus resistant environment.

## Data Availability

The raw data supporting the conclusions of this article will be made available by the authors, without undue reservation.

## Supplementary material

Supplementary Figure 1 Comparison of RAW264.7 versus Ubi_K48R cells infected with MNoV_S99. **(A)** Viral titers from RAW264.7 (parental cells) and RAW264.7-Ubi_K48R (Ubi_K48R) cells infected with MNoV_S99 (MOI 0.1, 16h) were determined from supernatants using the TCID_50_ titration method. **(B)** Ubi_K48R and the parental cells were infected with MNoV_S99 (MOI 0.1, 16h) or mock treated (θ) and stained with anti-dsRNA antibodies (shown in red). The YFP_Ubiquitin signal is shown in green. The nuclei were counterstained with DAPI (blue) in the merged images. **(C)** pIκBα, IκBα and ß -Tubulin expression levels were measured by western blot analysis from total proteins lysates from Ubi_K48R and the parental infected cells for 16h with MNoV_S99 (MOI 1) or mock infected.

Supplementary Figure 2 Comparison of Ubi_WT versus Ubi_K48R cells infected with MNoV_S99. Ubi_WT or Ubi_K48R cells were infected with MnoV_S99 (MOI 1, 16h) or mock treated (θ) and stained with anti-dsRNA antibodies (shown in red). The YFP_Ubiquitin signal is shown in green. The nuclei are counterstained with DAPI shown in blue in the merged images. Labelled cells were imaged using confocal spinning-disk microscope with a 40x objective.
