# Supplementary figures and images for: Impaired K48-polyubiquitination downmodulates mouse norovirus propagation
Source: Front Cell Infect Microbiol. 2025 May 6;15:1530166. doi: 10.3389/fcimb.2025.1530166 (PMC12089143; doi:10.3389/fcimb.2025.1530166)

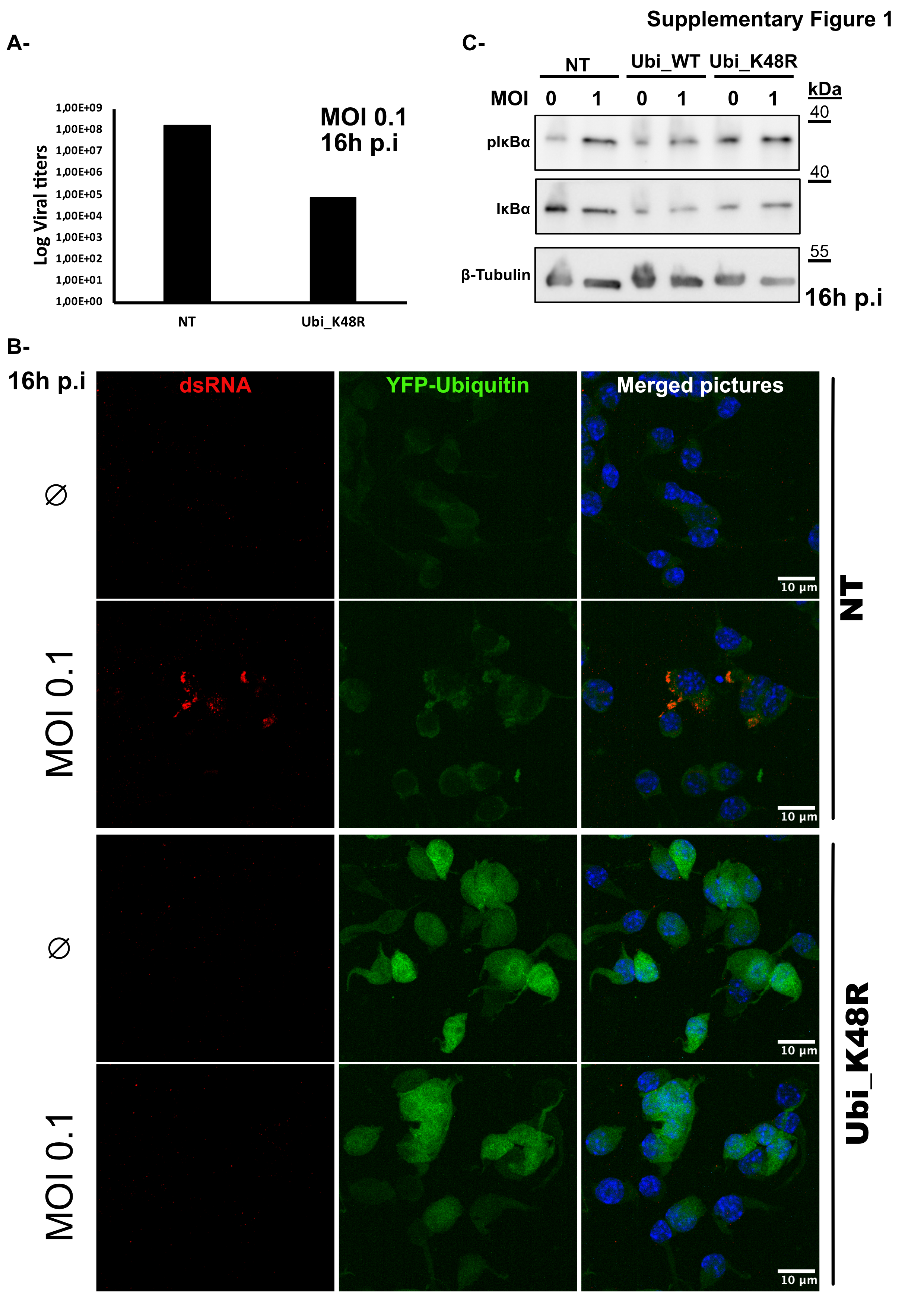

Supplement: Supplementary Figure 1 — Comparison of RAW264.7 versus Ubi_K48R cells infected with MNoV_S99. (A) Viral titers from RAW264.7 (parental cells) and RAW264.7-Ubi_K48R (Ubi_K48R) cells infected with MNoV_S99 (MOI 0.1, 16h) were determined from supernatants using the TCID50 titration method. (B) Ubi_K48R and the parental cells were infected with MNoV_S99 (MOI 0.1, 16h) or mock treated (θ) and stained with anti-dsRNA antibodies (shown in red). The YFP_Ubiquitin signal is shown in green. The nuclei were counterstained with DAPI (blue) in the merged images. (C) pIκBα, IκBα and ß -Tubulin expression levels were measured by western blot analysis from total proteins lysates from Ubi_K48R and the parental infected cells for 16h with MNoV_S99 (MOI 1) or mock infected. [file Image1.tiff]

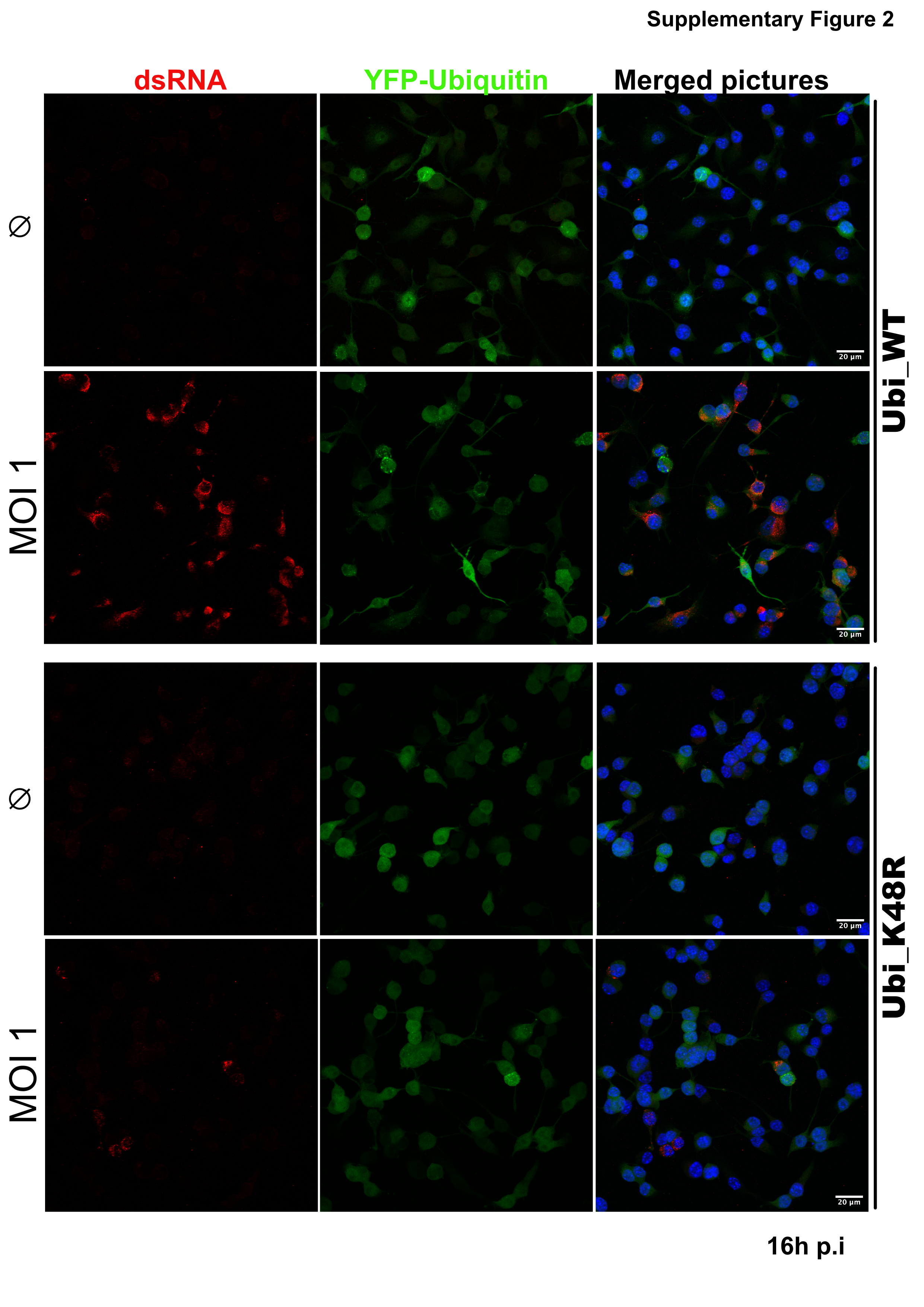

Supplement: Supplementary Figure 2 — Comparison of Ubi_WT versus Ubi_K48R cells infected with MNoV_S99. Ubi_WT or Ubi_K48R cells were infected with MnoV_S99 (MOI 1, 16h) or mock treated (θ) and stained with anti-dsRNA antibodies (shown in red). The YFP_Ubiquitin signal is shown in green. The nuclei are counterstained with DAPI shown in blue in the merged images. Labelled cells were imaged using confocal spinning-disk microscope with a 40x objective. [file Image2.tiff]
